# Detection of neuroinflammation before selective neuronal loss appearance after mild focal ischemia using [^18^F]DPA-714 imaging

**DOI:** 10.1186/s13550-018-0400-x

**Published:** 2018-06-08

**Authors:** Natsumi Miyajima, Miwa Ito, Takemi Rokugawa, Hitoshi Iimori, Sotaro Momosaki, Shigeki Omachi, Eku Shimosegawa, Jun Hatazawa, Kohji Abe

**Affiliations:** 1 Translational Research Unit, Biomarker R&D Department, Shionogi & Co., Ltd., Osaka, 5610825 Japan; 2 Department of Applied Chemistry and Analysis, Research Laboratory for Development, Shionogi & Co., Ltd., Osaka, Japan; 3 Department of medical affairs, Shionogi & Co., Ltd., Osaka, Japan; 4 Department of Molecular Imaging in Medicine, Osaka University Graduate School of Medicine, Osaka, Japan; 5 Department of Nuclear Medicine and Tracer Kinetics, Osaka University Graduate School of Medicine, Osaka, Japan; 6 PET Molecular Imaging Center, Osaka University Graduate School of Medicine, Osaka, Japan

**Keywords:** Mild ischemia, Translocator protein, Selective neuronal loss, [^18^F]DPA-714 binding

## Abstract

**Background:**

Translocator protein (TSPO) imaging can be used to detect neuroinflammation (including microglial activation) after acute cerebral infarction. However, longitudinal changes of TSPO binding after mild ischemia that induces selective neuronal loss (SNL) without acute infarction are not well understood. Here, we performed TSPO imaging with [^18^F]DPA-714 to determine the time course of neuroinflammation and SNL after mild focal ischemia.

**Results:**

Mild focal ischemia was induced by middle cerebral artery occlusion (MCAO) for 20 min. In MCAO rats without acute infarction investigated by 2, 3, 5-triphenyltetrazolium chloride (TTC) staining, in vitro ARG revealed a significant increase of [^18^F]DPA-714 binding in the ipsilateral striatum compared with that in the contralateral side at 1, 2, 3, and 7 days after MCAO. Increased [^18^F]DPA-714 binding was observed in the cerebral cortex penumbra, reaching maximal values at 7 days after MCAO. Activation of striatal microglia and astrocytes was observed with immunohistochemistry of ionized calcium binding adaptor molecule 1 (Iba1) and glial fibrillary acidic protein (GFAP) at 2, 3, and 7 days after MCAO. SNL was investigated with Nissl staining and neuronal nuclei (NeuN) immunostaining and observed in the ischemic core region of the striatum on days 3 and 7 after MCAO. We confirmed that total distribution volume of [^18^F]DPA-714 in the ipsilateral striatum was significantly increased at 2 and 7 days after MCAO using positron emission tomography (PET).

**Conclusions:**

[^18^F]DPA-714 binding measured with in vitro ARG was increased before SNL appeared, and this change was detected by in vivo PET. These findings suggest that TSPO PET imaging might be useful for detection of neuroinflammation leading to SNL after focal ischemia.

## Background

Stroke is the second most common cause of death worldwide, with approximately 80% of strokes attributed to occlusion of blood vessels [1]. In the severe middle cerebral artery occlusion (MCAO) rat model of stroke, the MCA is occluded for 60–120 min, and brain infarction of the whole striatum can be observed with magnetic resonance imaging (MRI) or 2,3,5-triphenyltetrazolium chloride (TTC) staining at 1 day after reperfusion [2, 3]. Neuroinflammation is widely accepted as a contributing factor in the pathophysiology of ischemic stroke and neuronal cell death [4]. Several studies have shown that microglia are rapidly and time-dependently activated after ischemia, with microglial activation reported to reflect the severity of ischemic damage [5, 6]. Microglial activation exerts a cytotoxic effect by releasing inflammatory mediators (including cytokines, chemokines, proteases, and reactive oxygen species) that trigger neuronal damage [7, 8]. Astrocyte activation also induces the release of large amounts of tumor necrosis factor-α, resulting in abundant glutamate and neurotoxic damage [9]. These previous findings suggest that acute neuroinflammation, including microglial and astrocytic activation, is a key component of the pathophysiological processes following cerebral ischemia and is related to the severity of resulting brain damage.

Mild ischemia that does not induce cerebral infarction has also been shown to induce activation of microglia and/or astrocytes [10, 11]. Fujioka et al. [10] reported that MCAO for 15 min induced microglial and astrocytic activation at 3 days after reperfusion, with loss of dorsolateral striatal neurons at 7 days. Further, striatal selective neuronal loss (SNL) can be induced by brief MCAO without infarction, but this includes microglia and astrocyte activation [12, 13]. In previous studies, we reported SNL at 7 days after 20-min MCAO, without acute brain infarction [14]. Taken together, these lines of evidence suggest that the activation of microglia and astrocytes after mild ischemia might be a key factor in SNL. In clinical settings, mild ischemia has been found to cause cognitive impairment in patients more than 1 year after an ischemic attack [15]. Mild ischemia also induces SNL, which is an important factor in the risk of later decline of brain function [10]. Predicting SNL could provide an opportunity for intervention using pharmacological or genetic means for post-stroke treatment.

Translocator protein (TSPO) is expressed in the outer membrane of mitochondria. Although TSPO is scarce under healthy circumstances, its expression is enhanced when astrocytes and microglia are activated [16]. It has been reported that the uptake of TSPO ligands increases when neuroinflammation is induced, with subsequent activation of microglia [17]. TSPO positron emission tomography (PET) imaging can be used to examine microglial activation in brain disease models and patients with neurological disorders [17–19]. Numerous TSPO ligands have been developed, exhibiting high affinity for TSPO and improved signal to noise ratios compared with [^11^C]PK11195, the first TSPO ligand to be studied. [^18^F]DPA-714 is one such TSPO ligand, with a high affinity for TSPO, demonstrated using ex vivo autoradiography (ARG) and in vivo PET of the brain in a rat model of inflammation [20–22]. The use of [^18^F]DPA-714 in patients with Alzheimer’s disease [23] and quantification of [^18^F]DPA-714 kinetics [24] have been reported, suggesting the feasibility of translating non-clinical findings into clinical practice. [^18^F]DPA-714 has been also used in animal models of stroke, and patients with stroke [25–30]. In a severe MCAO rat model involving occlusion for 120 min, Martín et al. [27] reported increased uptake of [^18^F]DPA-714 in the ipsilateral area from 4 to 30 days after reperfusion, reaching a maximal binding value on day 11. One study of a MCAO model involving occlusion for 60 min confirmed increased uptake of TSPO ligands, [^18^F]GE180 and [^11^C]PK11195, at 5 or 6 days after reperfusion using in vivo PET imaging [2]. These changes in TSPO binding after ischemia have been investigated in severe ischemia models involving acute brain infarction. However, to the best of our knowledge, previous studies have not applied TSPO imaging after mild focal ischemia without acute brain infarction.

In the current study, we investigated longitudinal changes of [^18^F]DPA-714 in vitro ARG in rats after mild focal ischemia with 20-min MCAO, which does not induce apparent brain infarction. In addition, neuronal loss and expression of ionized calcium binding adaptor molecule 1 (Iba1) and glial fibrillary acidic protein (GFAP) were also examined. Furthermore, PET imaging with [^18^F]DPA-714 was performed to confirm whether changes of TSPO binding observed using in vitro ARG can be detected noninvasively.

## Methods

### Animals

Seventy-six male Wistar rats were purchased from Japan Clea (Tokyo, Japan) and were 9–10 weeks old at the time of experiments. Rats were allowed free access to chow and tap water and were housed in a temperature-controlled room maintained on a 12-h light/dark cycle with lights on at 8:00 am. The experimental protocols were reviewed and approved by the Institutional Animal Care and Use Committee of Shionogi Research Laboratories and Osaka University Graduate School of Medicine (Osaka, Japan).

### Radioligand

[^18^F]DPA-714 was produced as described previously [21, 31, 32]. Briefly, a solution of 5 mg precursor in 0.7 mL anhydrous acetonitrile was added to the [^18^F]KF/kryptofix complex. The mixture was allowed to react for 10 min at 110 °C. The reaction mixture was then injected into COSMSIL C18 MS-II (20 × 250 mm) with MeCN/30 mM AcONH_4_ (50/50). The purified fraction was evaporated until dry, and the residue was dissolved in saline containing EtOH and polysorbate 80. The specific activity of the product was 51–212 GBq/μmol at the end of synthesis, and the radiochemical purity exceeded 99%.

### Mild focal cerebral ischemic model

The MCAO rat model was established as in our previous studies [14], with some modifications. Briefly, rats weighing approximately 300 g were initially anesthetized with 5% isoflurane and maintained under anesthesia with 2% isoflurane (room air, 1.5 L/min). Rats were placed on a hot plate (38 °C) during the operation to induce left MCAO. A 4–0 monofilament nylon suture thread (Natsume Seisakusho, Tokyo, Japan) coated with silicon (Heraseus, Kulzer, Germany) was inserted via the common carotid artery into the internal carotid artery, then into the circle of Willis. The suture thread was inserted 20 to 22 mm from the bifurcation of the common carotid artery. After the intraluminal suture thread had been positioned, the neck incision was closed with a silk suture. The duration of the occlusion period was 20 min. Neurological deficits (exhibited as right forepaw paralysis and Horner’s syndrome) were used as criteria for successful MCAO, as described previously [10]. The MCAO operation was successful for all 76 rats. Reperfusion was carried out by removing the intraluminal suture thread. Rats were selected by TTC staining and used for each study. Twenty of 36 operated rats were used for in vitro ARG and Nissl staining on days 1 (*n* = 5), 2 (*n* = 5), 3 (*n* = 4), and 7 (*n* = 6) after MCAO. Of 22 operated rats, 14 were used for immunohistochemistry at days 1 (*n* = 3), 2 (*n* = 4), 3 (*n* = 4), and 7 (*n* = 3) after MCAO. Of 18 operated rats, 14 were used for PET studies at days 2 (*n* = 10) and 7 (*n* = 4) after MCAO. The rats used in the PET experiment weighed 316 ± 27 g (day 2) and 336 ± 31 g (day 7).

### TTC staining

Rats were sacrificed by decapitation at 1, 2, 3, or 7 days after MCAO under deep anesthesia with isoflurane. Brains were quickly removed and divided into two blocks for TTC staining and other examinations. Anterior brains were chilled in ice-cold saline for 30 s and sliced into 2-mm-thick coronal sections with a tissue slicer. These sections were stained with 2.0% TTC (Sigma, St. Louis, MO, USA) solution at 37 °C for 10 min. Sections were imaged using a digital camera, and unstained areas were defined as the infarcted area. Rats with infarction were excluded from all studies.

### In vitro ARG

**Fig. 1:**
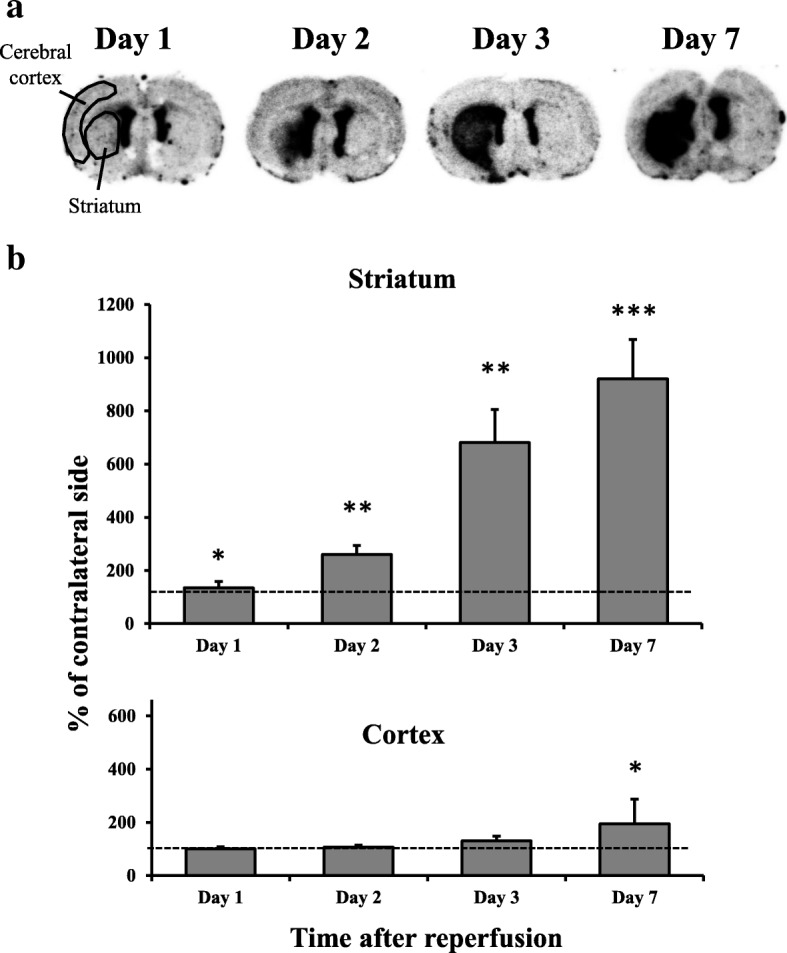
Results of [^18^F]DPA-714 binding in the brain after mild focal ischemia. **a** Typical autoradiograms of [^18^F]DPA-714 binding in the brain after mild focal ischemia. Coronal sections at the level of the striatum were prepared at 1, 2, 3, and 7 days after 20-min middle cerebral artery occlusion (MCAO). The left side of each image is the ipsilateral side. **b** Temporal changes in [^18^F]DPA-714 binding in the striatum and cortex after mild focal ischemia. Graphs show % of contralateral side, with values expressed as mean ± SD (day 1: *n* = 5, day 2: *n* = 5, day 3: *n* = 4, day 7: *n* = 6). Asterisks indicate significant differences compared with the contralateral side, *p < 0.05, **p < 0.01, ***p < 0.001

At 1, 2, 3, or 7 days after MCAO, the brain block was frozen in powdered dry ice. Coronal sections (20 μm) were prepared using a cryostat (CM3050S; Leica, Nussloch, Germany) and stored at − 80 °C until use. Sections were pre-incubated in 50 mM Tris-HCl buffer (pH 7.4) at 4 °C for 10 min, followed by 2 h incubation in the same buffer with 1–4 nM of [^18^F]DPA-714. Sections were then washed twice with fresh buffer for 5 min each wash, then dipped in distilled water at 4 °C. Dried sections were exposed to an imaging plate (BAS-SR; FUJIFILM, Tokyo, Japan) for 2 h. After exposure, plates were read with a bio-imaging analyzer system (FLA-7000; GE Healthcare Bio-Sciences, Uppsala, Sweden). To quantify radioactivity from autoradiograms, regions of interest (ROIs) were set as shown in Fig. 1a, and photo-stimulated luminescence values for each ROI (count/pixel^2^) were determined using Multi Gauge version 3.0 (FUJIFILM, Tokyo, Japan).

### Nissl staining

Coronal sections (10 μm) from the same animal used for in vitro ARG were stained with cresyl violet (Nissl staining). Briefly, sections were dehydrated using gradations of 30, 50, 75, 95, and 100% ethanol in water, and rehydrated using gradations of 95, 75, 50, and 30% ethanol in water. Sections were then stained with cresyl violet solution (0.25%) for 30 min at room temperature, dehydrated using gradations of 95 and 100% ethanol in water, then cleared with xylene. To determine neuronal loss, microscopic images were obtained using an Axioskop fluorescence microscope (Zeiss, Oberkochen, Germany). The number of neurons within a box measuring 4 × 10^4^ μm^2^ was counted from the contralateral and ipsilateral sides of the striatum and cerebral cortex. Data were obtained for two sections per animal from four to six rats.

### PET/computed tomography (CT) scans

At 2 and 7 days after MCAO, PET/CT scans were performed under isoflurane anesthesia (2–3%) using a small-animal PET/CT scanner (Pre-Clinical Imaging System LabPET-12; TriFoil Imaging Inc., Chatsworth, USA). A cannula was inserted into the tail vein for tracer injection, and into the femoral artery for arterial blood sampling. 0.5 mL of [^18^F]DPA-714 (injection dose: 19.8 ± 2.6 MBq, min./max. range: 16.7/25.7 MBq, specific activity at injection: 61.3 ± 49.5 GBq/μmol, min./max. range: 13.5/181 GBq/μmol) was injected for 30 s into the tail vein using a syringe pump (Legato-210, KD Scientific Inc. Holliston, MA, USA) in order to get the peak point of the plasma radioactivity for the quantitative PET analysis. For competition experiments, unlabeled (*R, S*)-PK11195 (3 mg/kg) was intravenously injected immediately before injection of [^18^F]DPA-714 at 2 days after MCAO. Emission data were collected for 60 min at the same time as the start of the tracer injection. After the PET scan, CT scans were performed to acquire anatomical information and to obtain data for attenuation correction of the PET images. After the PET/CT scans, the rats were sacrificed by decapitation, and brain tissue was collected for TTC staining. TTC staining was performed as described above to confirm the absence of infarction.

### [^18^F]DPA-714 blood metabolite analysis

During the PET scan, 11 blood samples of 0.1 mL were taken (10, 20, 30, 40, 50, 60, 90, 300, 900, 1800, and 3600 s) through the cannula of the femoral artery and centrifuged at 14,000 rpm (19,300×*g*) at 4 °C for 4 min. The radioactivity of plasma (10 μL) was measured using a gamma counter (2480 WIZARD^2^, PerkinElmer, Inc., Waltham, MA, USA). Metabolite analysis was performed at 90, 300, 900, 1800, and 3600 s as described previously [33]. Briefly, aliquots of plasma (10 μL) were retrieved, and extraction was performed using 30-μL acetonitrile. The mixture was vortexed and centrifuged at 14,000 rpm (19,300×g) at 4 °C for 4 min. The supernatant was removed and 10 μL from each sample was applied to silica gel thin-layer chromatography (TLC) plates (Merck KGaA, Darmstadt, Germany). A mixture of chloroform (90%), methanol (9%), and ammonium hydroxide (1%) was used as the eluent. The dried TLC plates were placed on an imaging plate for 1 h. After exposure, plates were read with FLA-7000 and the data was analyzed using ImageQuant (GE Healthcare UK Ltd., Amersham, England) to calculate the unmetabolized fraction of [^18^F]DPA-714 in the plasma, corrected for metabolites. The time course of the unmetabolized fraction of [^18^F]DPA-714 in the plasma was determined for individuals from the obtained unmetabolized fraction data using GraphPad Prism (version 5, GraphPad Software, Inc., La Jolla, CA, USA). To obtain the metabolite-corrected radioactivity concentration, the radioactivity concentration in the plasma was multiplied by the unmetabolized fraction at each point.

### PET/CT image analysis

CT images were reconstructed using a filtered back-projection method (512 slices), and list-mode data from the emission scan were reconstructed into 21 time frames (6 × 10, 4 × 60, 11 × 300 s) using the 3D-MLEM method with CT-based attenuation correction. CT and PET images were fused with PMOD image analysis software (version 3, PMOD Technologies Ltd., Zürich, Switzerland). The anatomical position of the striatum was checked and volumes of interest (VOIs; 4.2 mm × 4.5 mm × 3.8 mm sphere) were manually placed on the PET images. The radioactivity concentration was decay corrected and expressed as the percent injected dose per milliliter (%ID/mL). Standardized uptake values (SUVs) were calculated as the average radioactivity concentration for each VOI (%ID/mL) divided by body weight. The total distribution volume (*V*_T_) of [^18^F]DPA-714 in the ipsilateral and contralateral striatum was determined by Logan plot graphical analysis using the radioactivity of metabolite-corrected plasma as an input function. This analysis was performed using a PMOD kinetic modeling tool (PKIN).

### NeuN, Iba 1, and GFAP immunohistochemistry

At 1, 2, 3, or 7 days after reperfusion, brains were sliced into 4-mm-thick coronal sections. Sections were then fixed in 4% paraformaldehyde in phosphate buffered saline (PBS) for 2 h at room temperature and cryoprotected with 30% sucrose in 4 °C PBS overnight. Slabs were further sliced in coronal sections (12 μm thick) using a cryostat. For detection of microglia and astrocytes, sections were heated using a microwave oven at 600 W in 10 mM citrate buffer (pH 6) for 4 min to expose the antigen sites. After rinsing in PBS for 5 min, sections were incubated with blocking solution containing 3% bovine serum albumin at room temperature for 1 h, then incubated with mouse anti-NeuN monoclonal antibody (1:500; Merck Millipore, Darmstadt, Germany, Cat No. MAB377) for neurons, or rabbit anti-Iba1 polyclonal antibody (1:500; Wako, Osaka, Japan, Cat No. 019-19741) for microglia, or rabbit anti-GFAP polyclonal antibody (1:500; Dako, Glostrup Denmark, Cat No. Z0334) for astrocytes, at 4 °C overnight. After washing with PBS for 5 min, sections were reacted with Alexa Fluor 488-conjugated anti-mouse IgG antibody (1:500; Invitrogen, Carlsbad, CA, USA, Cat No. A-21202) for neurons, or Alexa Fluor 488-conjugated anti-rabbit IgG antibody (1:500; Invitrogen, Carlsbad, CA, USA, Cat No. A-21206) for microglia and astrocytes at room temperature for 1 h. After washing, sections were mounted using VECTASHIELD Mounting Medium with propidium iodide (Vector, Burlingame, CA, USA) and coverslipped. Immunohistochemical images were obtained using an Axioskop fluorescence microscope. The number of NeuN-positive cells was counted within a box measuring 4 × 10^4^ μm^2^ from the ipsilateral or contralateral dorsolateral striatum and cerebral cortex. The number of Iba 1-positive cells was counted within a box measuring 4 × 10^4^ μm^2^ from the ipsilateral or contralateral dorsolateral striatum and cerebral cortex. For GFAP staining, each section was manually binarized using ImageJ software (National Institutes of Health, Bethesda, Maryland, USA, http://imagej.nih.gov/il/) and the pixel count of a GFAP-positive area within a box measuring 4 × 10^4^ μm^2^ from the ipsilateral or contralateral dorsolateral striatum and cerebral cortex was determined. Data were analyzed under the same conditions between the ipsilateral and contralateral sides and were obtained for one or two sections per animal, from three to four rats.

### Statistical analysis

All results are expressed as mean ± SD. Comparisons between ipsilateral and contralateral sides were analyzed using paired *t*-tests. A *p* value of < 0.05 was considered to indicate statistical significance.

## Results

### In vitro ARG of [^18^F]DPA-714

[^18^F]DPA-714 binding was measured in the MCAO rats using ARG. Autoradiographic images show accumulation in the ipsilateral striatum of MCAO rats at 1, 2, 3, and 7 days after MCAO was obviously higher than that in the contralateral striatum (Fig. 1a). As shown in Fig. 1b, the radioactivity concentration significantly increased in the ipsilateral striatum to 134% (day 1), 260% (day 2), 681% (day 3), and 920% (day 7) of the contralateral side. On day 7, radioactivity concentration significantly increased in the ipsilateral cortex to 194% of the contralateral side. In contrast, there were no changes in the ipsilateral cortex on days 1, 2, and 3.

### TTC stains

**Fig. 2:**
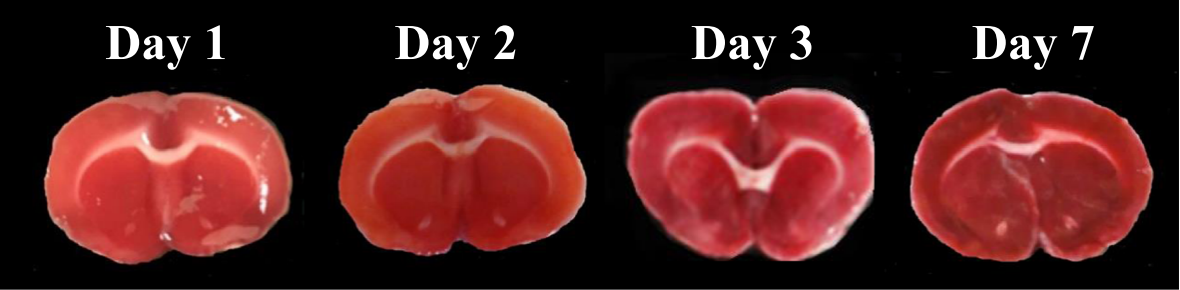
Typical 2,3,5-triphenyltetrazolium chloride (TTC)-stained images in the brain after mild focal ischemia. Coronal sections at the level of the striatum were prepared at 1, 2, 3, and 7 days after 20-min middle cerebral artery occlusion (MCAO). The left side of each image is the ipsilateral side

No clear differences between the ipsilateral and contralateral side of the brain were found at any of the time points investigated in TTC staining following 20 min MCAO as shown in Fig. 2. Twenty-eight of 76 MCAO rats exhibited an infarct (pale areas versus non-injured deep red colored tissue). The subsequent experiments were performed using MCAO rats without pale areas in the ipsilateral brain.

### Neuronal loss

**Fig. 3:**
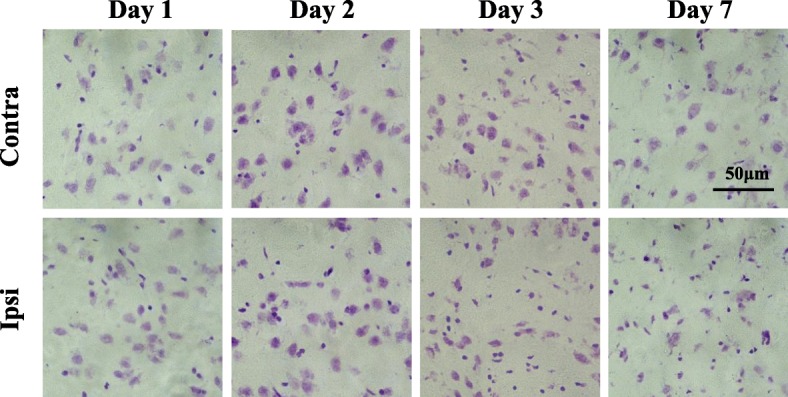
Typical Nissl staining images in the dorsal striatum after mild focal ischemia. The upper panels are the images of contralateral side striatum, and the bottom panels are the images of ipsilateral side striatum at 1, 2, 3, and 7 days after 20-min middle cerebral artery occlusion (MCAO). Scale bar, 50 μm. Contra, contralateral side; Ipsi, ipsilateral side

Nissl staining images are shown in Fig. 3. The number of neurons within 4 × 10^4^ μm^2^ of the contralateral or ipsilateral striatum is shown in Table 1. There were no changes in the contralateral striatum of MCAO rats at any point. The number of stained cells in the ipsilateral striatum decreased to 73% (26 ± 3 vs. 19 ± 4 cells) and 58% (24 ± 2 vs. 14 ± 5 cells) of the contralateral side on days 3 and 7, respectively, although no significant changes were observed in the ipsilateral striatum on days 1 and 2. The number of stained cells did not change in the cerebral cortex in MCAO rats at any point.

**Table 1:** Temporal changes in Nissl-stained cells in the striatum and cortex after mild focal ischemia

Time after reperfusion	Striatum		Cortex	
	Ipsi	Contra	Ipsi	Contra
Day 1	25 ± 2	26 ± 2	26 ± 4	29 ± 5
Day 2	27 ± 1	29 ± 3	27 ± 2	28 ± 3
Day 3	19 ± 4*	26 ± 3	28 ± 2	28 ± 2
Day 7	14 ± 5**	24 ± 2	26 ± 1	25 ± 3

The data are expressed as mean ± SD (day 1: *n* = 5, day 2: *n* = 5, day 3: *n* = 4, day 7: *n* = 6) of the number of cells per field of view (4 × 10^4^ μm^2^). Asterisks indicate significant differences compared with the contralateral side, *p < 0.05, **p < 0.01. MCAO, middle cerebral artery occlusion; Contra, contralateral side; Ipsi, ipsilateral side

**Fig. 4:**
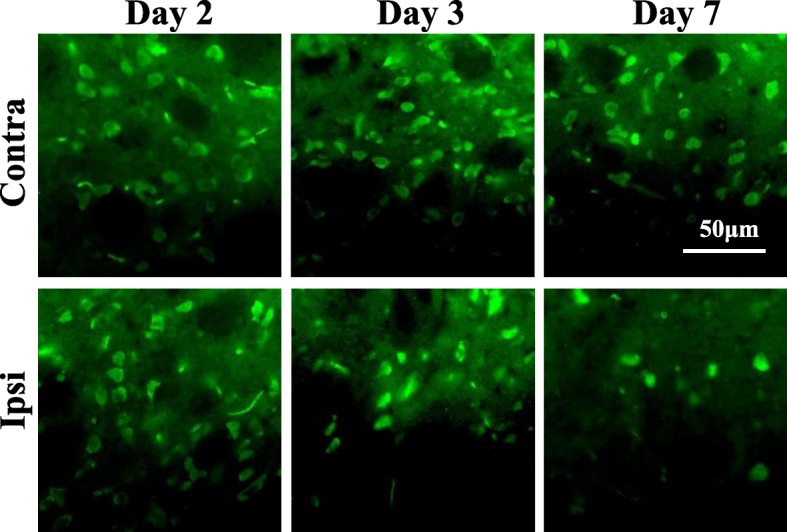
Typical NeuN immunostaining images in the dorsal striatum after mild focal ischemia. The upper panels are the images of contralateral side striatum, and the bottom panels are the images of ipsilateral side striatum at 2, 3, and 7 days after 20-min middle cerebral artery occlusion (MCAO). Scale bar, 50 μm. Contra, contralateral side; Ipsi, ipsilateral side

NeuN immunohistochemistry was performed to confirm the results of Nissl staining at days 2, 3, and 7. NeuN-immunostained images are shown in Fig. 4. The number of neurons within 4 × 10^4^ μm^2^ of the contralateral or ipsilateral striatum is shown in Table 2. The number of NeuN-positive cells in the ipsilateral striatum decreased to 44% (24 ± 2 vs. 11 ± 1 cells) and 25% (24 ± 2 vs. 6 ± 2 cells) of the contralateral side on days 3 and 7, respectively, although no significant changes were observed in the ipsilateral striatum on day 2. The number of NeuN-positive cells did not change in the cerebral cortex in MCAO rats at any point.

**Table 2:** Temporal changes in NeuN-positive cells in the striatum and cortex after mild focal ischemia

Time after reperfusion	Striatum		Cortex	
	Ipsi	Contra	Ipsi	Contra
Day 2	26 ± 2	27 ± 0	27 ± 3	26 ± 2
Day 3	11 ± 1*	24 ± 2	25 ± 3	27 ± 3
Day 7	6 ± 2**	24 ± 2	26 ± 2	26 ± 2

The data are expressed as mean ± SD (day 2: *n* = 3, day 3: *n* = 3, day 7: *n* = 3) of the number of NeuN-positive cells per field of view (4 × 10^4^ μm^2^). Asterisks indicate significant differences compared with the contralateral side, *p < 0.05, **p < 0.01. MCAO, middle cerebral artery occlusion; Contra, contralateral side; Ipsi, ipsilateral side

### Iba1 and GFAP immunohistochemistry

**Fig. 5:**
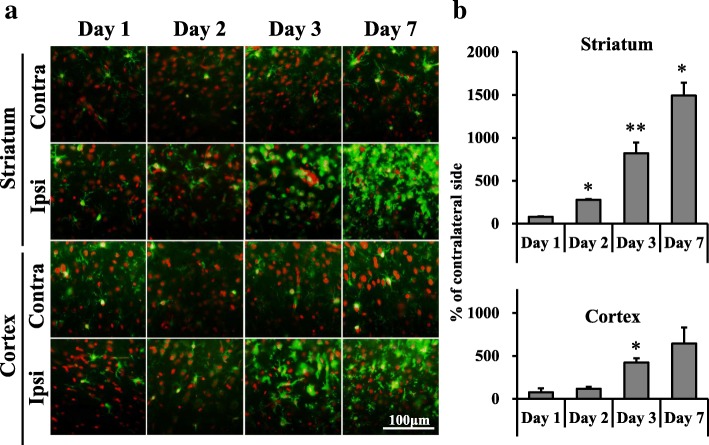
Immunostaining with Iba1 in the dorsal striatum and cerebral cortex after mild focal ischemia. **a** Merged immunostaining images are showed with Iba1 (green) and PI (red) at 1, 2, 3, and 7 days after 20-min middle cerebral artery occlusion (MCAO). Scale bar, 100 μm. Contra, contralateral side; Ipsi, ipsilateral side. **b** Graphs show % of contralateral side, with values expressed as mean ± SD (day 1: *n* = 3, day 2: *n* = 4, day 3: *n* = 4, day 7: *n* = 3). Asterisks indicate significant differences compared with the contralateral side, *p < 0.05, **p < 0.01

**Fig. 6:**
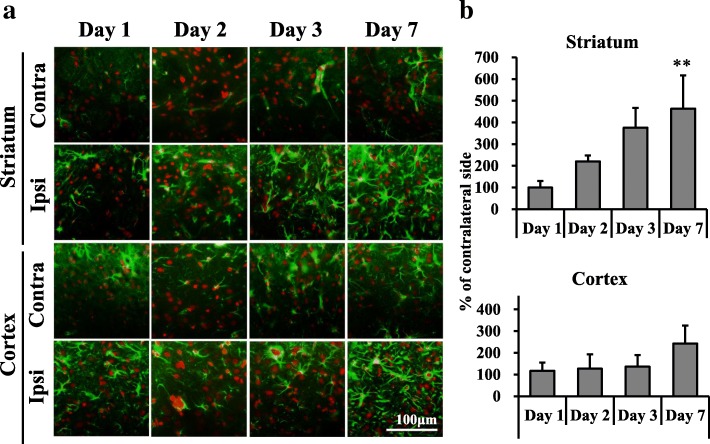
Immunostaining with GFAP of the dorsal striatum and cerebral cortex after mild focal ischemia. **a** Merged immunostaining images were captured with GFAP (green) and PI (red) at 1, 2, 3, and 7 days after 20-min middle cerebral artery occlusion (MCAO). Scale bar, 100 μm. Contra, contralateral side; Ipsi, ipsilateral side. **b** Graphs show % of the contralateral side, with values expressed as mean ± SD (day 1: *n* = 3, day 2: *n* = 3, day 3: *n* = 3, day 7: *n* = 3). Asterisks indicate significant differences compared with the contralateral side, **p < 0.01

Iba1-immunostained images are shown in Fig. 5a. The number of Iba1-positive cells in the ipsilateral striatum significantly increased to 278% (day 2), 819% (day 3), and 1492% (day 7) (Fig. 5b). There were no changes in the ipsilateral striatum on day 1. In the ipsilateral cerebral cortex, the number of Iba1-positive cells increased to 424% on day 3. There were no changes in the ipsilateral cortex on days 1, 2, and 7. GFAP-immunostained images are shown in Fig. 6a. The ratio of the GFAP-positive area in the ipsilateral striatum increased to 221% (day 2) and 376% (day 3) (Fig. 6b), but these changes were not significant. On day 7, the GFAP-positive area significantly increased to 464% (day 7). The ipsilateral to contralateral ratio of the GFAP-positive area in the striatum increased over time, reaching a significant level only at day 7 post-stroke (464%, Fig. 6b). There were no changes in the ipsilateral striatum on day 1. In the ipsilateral cerebral cortex, the ratio of the GFAP-positive area increased on day 7, but this change was not significant. The ratio of the GFAP-positive area was not altered in the ipsilateral cerebral cortex on days 1, 2, and 3.

### [^18^F]DPA-714 PET

**Fig. 7:**
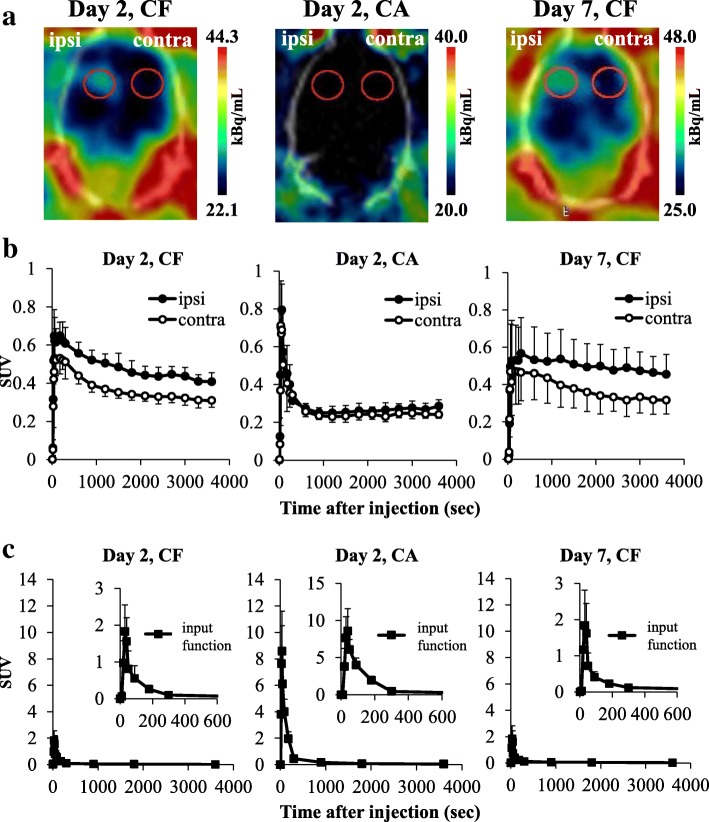
Typical [^18^F]DPA-714 PET images and time activity curves in the striatum. **a** Typical horizontal summed PET images of 20–60-min scans at days 2 and 7 after 20-min middle cerebral artery occlusion (MCAO). The left side of each image is the ipsilateral side. These images including the VOIs (red circles) show screen captures from PMOD. **b** The time activity curves of the VOI placed on the ipsilateral or the contralateral striatum of 20-min MCAO rats injected with [^18^F]DPA-714 at day 2 (CF: *n* = 5) and day 7 (CF: *n* = 4) or [^18^F]DPA-714 following unlabeled (*R, S*)-PK11195 (3 mg/kg) at day 2 (CA: *n* = 5). **c** Metabolite-corrected plasma radioactivity (input function) of 20-min MCAO rats injected with [^18^F]DPA-714 at day 2 (CF, *n* = 5) and day 7 (CF: *n* = 4) or [^18^F]DPA-714 following unlabeled (*R, S*)-PK11195 (3 mg/kg) at day 2 (CA: *n* = 5). Graphs show SUV, with values expressed as mean ± SD. *CF: A group of rats was injected [^18^F]DPA-714 only. **CA: A group of rats was injected [^18^F]DPA-714 and an excess of unlabeled (*R, S*)-PK11195

We analyzed data from rats without infarction, confirmed with TTC staining. The coronal and horizontal brain summed images of PET scans (20–60 min) at days 2 and 7 are shown in Fig. 7a. Substantial accumulation was seen in the ipsilateral striatum. Time activity curves (TACs) of SUVs in the ipsilateral and contralateral striatum at days 2 and 7 are shown in Fig. 7b. [^18^F]DPA-714 uptake in the striatum reached a peak value at 1–5 min after injection and slowly disappeared. On day 2, SUV at 20–60 min after injection revealed that [^18^F]DPA-714 uptake was 37% higher in the ipsilateral striatum (SUV = 0.46) compared with the contralateral striatum (SUV = 0.34). On day 7, SUV at 20–60 min after injection revealed that [^18^F]DPA-714 uptake was 44% higher in the ipsilateral striatum (SUV = 0.48) compared with the contralateral striatum (SUV = 0.33). As shown in Fig. 7c, the unmetabolized [^18^F]DPA-714 uptake in plasma (input function) reached a peak value at around 40 s after injection and immediately disappeared. As shown in Table 3, *V*_T_ values of the ipsilateral striatum (12.9 ± 1.9) were significantly higher than those on the contralateral side (9.7 ± 1.3) on day 2. *V*_T_ values of the ipsilateral striatum (12.4 ± 2.5) were also significantly higher than those on the contralateral side (7.8 ± 0.8) on day 7. The *V*_T_ ratio (ipsilateral/contralateral stratum) was higher on day 7 (ratio: 1.6) than on day 2 (ratio: 1.3).

**Table 3:** *V*_T_ values in the striatum after mild focal ischemia

Time after reperfusion	Ipsi	Contra	Ratio (Ipsi/Contra)
Day 2	12.9 ± 1.9*	9.7 ± 1.3	1.3 ± 0.2
Day 7	12.4 ± 2.5*	7.8 ± 0.8	1.6 ± 0.2

The data are expressed as mean ± SD (day 2: *n* = 5, day 7: *n* = 4) of the *V*_T_ values. Asterisks indicate significant differences compared with the contralateral side, *p < 0.05. MCAO, middle cerebral artery occlusion; Contra, contralateral side; Ipsi, ipsilateral side

Competition experiments at day 2 revealed that SUVs of [^18^F]DPA-714 in the ipsilateral and contralateral striatum decreased to the same level by an excess of unlabeled (*R, S*)-PK11195 (Fig. 7b). This finding indicates that increased accumulation of [^18^F]DPA-714 in the ipsilateral striatum was TSPO-specific. [^18^F]DPA-714 uptake in the striatum reached a peak value at around 60 s after injection and immediately washed out (Fig. 7c). The initial uptake of [^18^F]DPA-714 (around 60 s) did not change between the ipsilateral and contralateral striatum. This data suggests that the blood flow was not affected by MCAO.

Following the injection of [^18^F]DPA-714, the proportion of radioactivity in the plasma that was due to the presence of the parent compound decreased over time. The proportions of unmetabolized [^18^F]DPA714 were 99, 97, 84, 58, and 29% at 90, 300, 900, 1800, and 3600 s after tracer injection, respectively. In contrast, the proportions of unmetabolized [^18^F]DPA714 were 99, 72, 39, 25, and 7% at 90, 300, 900, 1800, and 3600 s, respectively, after tracer with an excess of unlabeled (*R, S*)-PK11195 injection.

## Discussion

In cases of severe ischemia in which acute infarction is induced, a number of studies have reported that increased TSPO binding after ischemia with infarction can be detected with PET and ARG [2, 3, 27, 28]. Pulagam et al. [28] reported that PET imaging with [^18^F]DPA-714 showed a progressive increase in the ischemic brain hemisphere in 90-min MCAO rats during the first week, peaking at day 7. They also reported that increased [^18^F]DPA-714 binding occurred over 4 weeks after induction of ischemia in severe ischemia models with MCAO. In severe focal ischemia models, necrosis response can be induced in the ischemic core, and subsequent increased TSPO binding can also be induced. In the case of mild ischemia without acute infarction, the process of neuronal cell death is thought to differ from severe ischemia and is known as SNL. Few previous experiments have examined the effects of changes in TSPO binding on the process of SNL. In the present study, we used a rat model of short durations of ischemia using 20-min MCAO, which did not induce apparent cerebral infarction. The current findings confirmed that a mild ischemia model without acute infarction induced SNL in the ischemic core at 3 and 7 days after MCAO, while glial activation occurred at 2, 3, and 7 days after MCAO. Fujioka et al. [10] reported that microglial and astrocytic activation occurred from 3 days after 15-min MCAO that did not induce cerebral infarction, while SNL occurred at 7 days. In both cases, glial activation was induced prior to SNL in the striatum in the mild MCAO model. Hughes et al. [34] also reported a correlation between SNL and microglial activation in the non-infarcted cortex at day 14 after 45-min MCAO. These previous reports strongly suggest that microglial activation is related to SNL.

In our mild ischemic model, increased [^18^F]DPA-714 binding in the ipsilateral striatum was observed from day 1 after 20-min MCAO. SNL in the ipsilateral striatum was observed on days 3 and 7 after MCAO. These findings indicate that TSPO responded to mild ischemia before SNL had occurred. This increase of [^18^F]DPA-714 binding in the present study is likely to be due to microglial and astrocytic activation. Both Iba1- and GFAP-immunostained cells were increased in the ipsilateral striatum at 2 days after MCAO, as shown in Figs. 4 and 5, although the increase in GFAP-positive cells was not significant. TSPO-Iba1 and TSPO-GFAP double immunohistochemistry may be useful for identifying the cell population expressing the highest levels of TSPO in this model. Additionally, a substantial accumulation of [^18^F]DPA-714 was also observed in the cortex on day 7. Nonetheless, SNL in the cortex was not observed until 7 days after 20-min MCAO. Modo et al. [35] reported that 15-min four-vessel occlusion (4-VO) induced selective damage in the hippocampal CA1 region, whereas 25-min 4-VO produced greater cell loss in CA1 with more variability in CA2, CA3, striatum, and cortex. Among the regions analyzed, neuronal cell death was mildest in the cortex. Hippocampal CA1 pyramidal neurons and striatal medium-sized neurons are highly vulnerable to transient cerebral ischemia [36]. Therefore, mild ischemia for 20 min might trigger increased [^18^F]DPA-714 binding, and activation of microglia and astrocytes, although vulnerability to neuroinflammation such as activation of microglia might differ between the cortex and striatum. Pulagam et al. [28] reported that [^18^F]DPA-714 binding in 90-min MCAO model rats was significantly increased not only in the striatum but also in the cortex, thalamus, and cerebellum of the ischemic brain hemisphere at 3 days after reperfusion. Thus, the speed of spread to the penumbra may be slower in the mild ischemia model compared with the severe model. Accumulating evidence indicates that transient cerebral ischemia can induce delayed neurodegeneration and apoptosis [36, 37]. Prolonged extracellular glutamate and aspartate levels are reported to be key factors contributing to neuronal degeneration after cerebral ischemia [38]. These excitatory amino acids induce overstimulation of glutamate receptors, such as NMDA or AMPA, and lead to increased calcium and ultimately neuronal death [39]. Arlicot et al. [40] also reported that quinolinic acid, an endogenous NMDA receptor agonist, induced increased TSPO binding and activation of microglia and astrocytes. Such glutamatergic excitotoxicity is thought to be a trigger for increased TSPO binding and SNL after mild ischemia.

In the current study, we performed in vivo PET imaging with [^18^F]DPA-714 and detected an increase in *V*_T_ values in the ipsilateral striatum at 2 and 7 days after MCAO without acute infarction. Injection of unlabeled (*R, S*)-PK11195 decreased accumulation of [^18^F]DPA-714 in the ipsilateral striatum, demonstrating that the enhanced accumulation of [^18^F]DPA-714 in the ipsilateral striatum was TSPO-specific. The *V*_T_ ratio (ipsilateral / contralateral striatum) of [^18^F]DPA-714 on day 7 was higher compared to that on day 2, although the increased ratio (day 7/day 2) of PET was smaller than that of ARG. One possible explanation of this difference is related to differences in the ROI studied. In in vitro experiments, we evaluated the ROIs in cross sections where the changes were clear as shown in Fig. 1. In contrast, the VOIs of in vivo PET were sterically defined as slightly larger (as shown in Fig. 7a) because CT images were insufficient for defining the anatomical position of the striatum. Thus, the VOIs of in vivo PET might contain a relatively large area of normal tissues that was not affected by ischemia compared to the ROIs of in vitro ARG. Moreover, because the resolution of PET imaging is substantially lower than the resolution of in vitro ARG, a partial volume effect (spillover) would be expected to significantly affect PET quantification (under-estimation), potentially contributing to the lower ipsilateral- to contralateral ratio. Another potential explanation is that the result was caused by differences in the ratio of specific binding between in vitro ARG and in vivo PET. Our preliminary data showed the percentage of specific binding of [^18^F]DPA-714 with in vitro ARG might be more than 90%. In contrast, as shown in Fig. 7b, the SUV value at 60 min after [^18^F]DPA-714 administration was 0.31 (CF: A group of rats was injected [^18^F]DPA-714 only) and 0.24 (CA: A group of rats was injected [^18^F]DPA-714 and an excess of unlabeled (*R, S*)-PK11195). This result indicated that the percentage of specific binding was approximately 30% in the contralateral striatum. Martin et al. [27] also reported that [^18^F]DPA-714 binding in the contralateral striatum was slightly decreased by injecting an excess of either PK11195 or DPA-714 20 min after tracer injection, indicating that specific binding in rat brain was low. However, in clinical studies, changes in [^18^F]DPA-714 binding after mild ischemia without acute infarction can be detected by PET because of the size of the human brain, and because VOIs can be defined in an anatomically accurate position using MRI. As for increased uptake of [^18^F]DPA-714 in the ipsilateral striatum after MCAO using PET, whether disruption of the brain blood barrier (BBB) has been induced by ischemia is important. The BBB disruption after severe ischemia has been reported in an MCAO model [41]. The infiltrated macrophages by the BBB disruption are potential sources of the TSPO expression in addition to the activated microglia, and it causes the overestimation of [^18^F]DPA-714 uptake. In the present study, the uptake of both sides of the striatum at 1 min after [^18^F]DPA-714 injection was no different in the competition study. These data suggest that the severe BBB disruptions which influence the accumulation of [^18^F]DPA-714 were not induced in our MCAO models. However, the further study on the BBB disruption will be needed to accurately interpret the present results in mild ischemia.

The most important novel finding in the current study was that TSPO binding with [^18^F]DPA-714 was significantly increased in the ipsilateral striatum before SNL appeared in the 20-min MCAO model. These findings suggest that mild cerebral ischemia can trigger neuroinflammation leading to SNL after mild ischemia, even if ischemia produces no acute cerebral infarction. SNL in the penumbra area, such as the cortex, also appeared to be involved in the process of infarction extension in the severe MCAO model. The process of SNL in the penumbra might be similar to the core area in the mild MCAO model, and the role of neuroinflammation might contribute to the extension of infarction in severe stroke. In the current study, increased TSPO binding was detected using in vivo PET with [^18^F]DPA-714 before SNL appeared. Therefore, the current findings suggest that TSPO imaging might be useful for detection of neuroinflammation leading to SNL after mild ischemia as well as severe ischemia. In particular, mild ischemia is considered to induce delayed cognitive impairment from several months to years later [42]. These cognitive disruptions are considered to be caused by SNL. Therefore, early detection of neuroinflammation leading to SNL after ischemia could be important for predicting pathophysiology and determining pharmacological therapy.

## Conclusions

[^18^F]DPA-714 binding determined with in vitro ARG was increased before SNL appeared, and this change could also be detected noninvasively using in vivo PET. These findings suggest that TSPO PET imaging might be useful for the detection of neuroinflammation leading to SNL after focal ischemia.
